# A snapshot on current approaches to lymphadenectomy in liver resection for intrahepatic cholangiocarcinoma: results from an international survey

**DOI:** 10.1007/s13304-024-01852-0

**Published:** 2024-05-07

**Authors:** Carlo Sposito, Marianna Maspero, Alessandro Cucchetti, Vincenzo Mazzaferro

**Affiliations:** 1 HPB Surgery and Liver Transplantation Unit, Fondazione, IRCCS Istituto Nazionale Tumori di Milano, Via Venezian 1, 20133 Milan, Italy; 2 Department of Oncology and Hemato-Oncology, University of Milan, Milan, Italy; 3 Department of Medical and Surgical Sciences, DIMEC, Alma Mater Studiorum, University of Bologna, Bologna, Italy; 4 Morgagni, Pierantoni Hospital, Forlì, Italy

**Keywords:** Intrahepatic Cholangiocarcinoma, Lymphadenectomy, Liver resection, Survey

## Abstract

The use of lymphadenectomy (LND) during resection of intrahepatic cholangiocarcinoma (ICC) is still debated, leading to differing practices in different centers and countries. The aim of this study was to assess such differences. A survey on LND for ICC was distributed to the members of the International Hepato-PancreatoBiliary Association (IHPBA) and the Italian Chapter of IHPBA (AICEP). Two-hundred thirty-four surgeons completed the survey (88% males; median age 46 years). Preoperative nodal staging was deemed mandatory/very important by 65%. Adequate LND was defined as hepatoduodenal ligament LND by 33%, LND at specific nodal stations by 28% and retrieval of > 5 nodes by 28%. The decision to perform LND was influenced by comorbidities (48%), chronic liver disease (38%) and satellitosis (32%). Most participants modify perioperative management in case of clinically positive nodes, 50% stating they would give neoadjuvant therapy. The role of LND in clinically node negative disease was adequate staging for 88%, survival benefit for 50.5% and clinical trials eligibility for 18.5%. Our survey confirms heterogeneity in the evaluation of role and extent of LND for ICC, how this relates to subjective perception of importance of LND, and need of a systematic approach in this area.

**Supplementary Information:**

The online version contains supplementary material available at 10.1007/s13304-024-01852-0.

## Introduction

Surgery is the only currently available curative treatment option for resectable intrahepatic cholangiocarcinoma (ICC), with 5-year overall survival between 25 and 40% [1, 2]. Recent advances have been made in the treatment of resectable ICC, both from the technical standpoint with the diffusion of minimally invasive surgery [3], and from the oncological standpoint with the introduction of more effective systemic therapies that may be used in the neoadjuvant and adjuvant setting [4, 5]. Despite these improvements, post-resection recurrence remains as high as 50–70% [6, 7], and different aspects of the surgical management of ICC are still controversial. One of these aspects is lymphadenectomy (LND), including whether it should be systematically performed and, if so, what is the ideal extent of LND and how adequate LND should be defined.

Nodal metastases, which may be present in up 40% of cases, are some of the strongest predictors of poor prognosis after curative intent surgery [8]. The preoperative assessment of nodal metastases is not standardized, and may miss positive lymph nodes in over one third of cases [9, 10]. Current guidelines, including European Society of Medical Oncology (ESMO) and American Joint Committee on Cancer (AJCC) [11, 12], recommend LND in all resections with curative intent for ICC, with the AJCC recommending the retrieval of at least six lymph nodes to ensure accurate staging. However, the role and usefulness of LND are debated by clinicians. Although retrospective evidence suggests a survival benefit both in clinically negative and clinically positive cases with adequate LND [10, 13], those results have not been validated by randomized controlled trials. While LND certainly plays a role in adequate staging, it has also been associated with significantly increased morbidity [14, 15], an important deterrent in view of an unclear prognostic benefit.

Since consensus is lacking, the surgical management of ICC varies greatly among different countries and centers. In this study, we report the results of a worldwide survey conducted to explore the current trends and attitudes toward LND in clinically negative and clinically positive cases, as well as preoperative assessment, patient selection, minimally invasive surgery, and neoadjuvant therapy.

## Methods

### Survey

This study was performed and reported according to the Checklist for Reporting Results of Internet E-Surveys (CHERRIES) [16]. During February 2023, a closed-survey (i.e. only accessible through invitation) was distributed via email to the members of the International Hepato-Pancreato-Biliary Association (IHPBA) and the members of Associazione Chirurgia Epato-bilio-Pancreatica (AICEP, Italian chapter of the IHPBA). The survey was composed of 29 questions on demographics, annual volume of surgeries for ICC, preoperative evaluation of nodal status, definition of adequate lymphadenectomy, and perioperative management of ICC (Appendix 1). The invitation email provided participants with details regarding the survey’s subject, the research team involved, and its objectives. Additionally, it outlined the estimated duration (approximately 5 min). Participants received no reminders. The survey was closed end of June 2023. The total number of invited participants, the number of participants who opened the email and the response rates were calculated. IP addresses or cookies were used to prevent multiple responses by the same individual and were deleted after the survey was closed. The survey was performed using REDCap electronic data capture tools hosted at the Institutional server of the IRCCS Istituto Nazionale dei Tumori (Milan, Italy) [17], and tested for usability and technical functionality. All submission were nominal. Submissions could be modified until the end of the survey collection period. Institutional Review Board approval was not requested since no patients were involved and informed consent was implied when participants completed the survey.

### Statistical analysis

The dataset was manually reviewed for entry errors. Incomplete submissions were included in the analysis if more than 50% of the survey had been completed and truncated at the last completed subsection.

Categorical variables were expressed as number and percentage, while numerical variables as median and interquartile range (IQR). We performed the following subgroup analyses: (1) East versus West; (2) surgeons from high volume (≥ 10 liver resections for ICC per year) vs low volume (< 10 liver resection for ICC per year) centers; (3) perceived importance of pre-nodal staging (very important/mandatory vs important/slightly important/not important at all). Categorical variables were compared using Fisher’s exact test, while numerical variables with Student’s t test. All analyses were two-sided. All analyses were performed in IBM SPSS Statistics for Microsoft Windows 24.0th Edition (IBM Corp., Armonk, NY, USA).

## Results

### Participants

The survey was sent to 3138 members, and the email was opened by 2087 members. The participants included in the analysis were 234, 11.2% of all surgeons who were administered the survey. The number of complete surveys was 208 (89%), while 26 (11%) participants submitted at least 50% of the survey. Table 1 reports the demographic characteristics of the participants, the volume of their center and the center’s routine assessment of specific mutations for ICC. Participants from high volume centers (> 10 liver resections for ICC per year) were 47%. Supplementary Fig. 1 shows the country of origin of the participants: the majority came from European countries (133, 57%), followed by the United States (36, 15%). Patients from Eastern countries were 27 (11%).

**Table 1:** Baseline characteristics of participants and Centers

Variable	*N* (%) or median (IQR)
Male sex	207 (88%)
Age (years)	46 (39–55)
Liver resections for ICC performed yearly	
0–5	46 (19.7)
5–10	78 (33.3)
10–20	67 (28.6)
20–40	28 (12.0)
> 40	15 (6.4)
Geographical area	
Europe	133 (56.8)
United States	36 (15.4)
Central/South America	30 (12.8)
Asia	27 (11.5)
Africa	8 (3.4)
Routine assessment of KRAS mutations	
Yes	84 (35.9)
No	55 (23.5)
In specific situations	89 (38.0)
Not available at my Center	4 (1.7)
Routine assessment of FGFR mutations	
Yes	66 (28.2)
No	69 (29.5)
In specific situations	90 (38.5)
Not available at my Center	7 (2.9)
Routine assessment of other specific mutations	
Yes	62 (26.5)
No	154 (65.8)
Not available at my Center	14 (5.9)

*ICC* intraepatic cholangiocarcinoma, *IQR* interquartile range

### Preoperative evaluation of nodal status

**Fig. 1:**
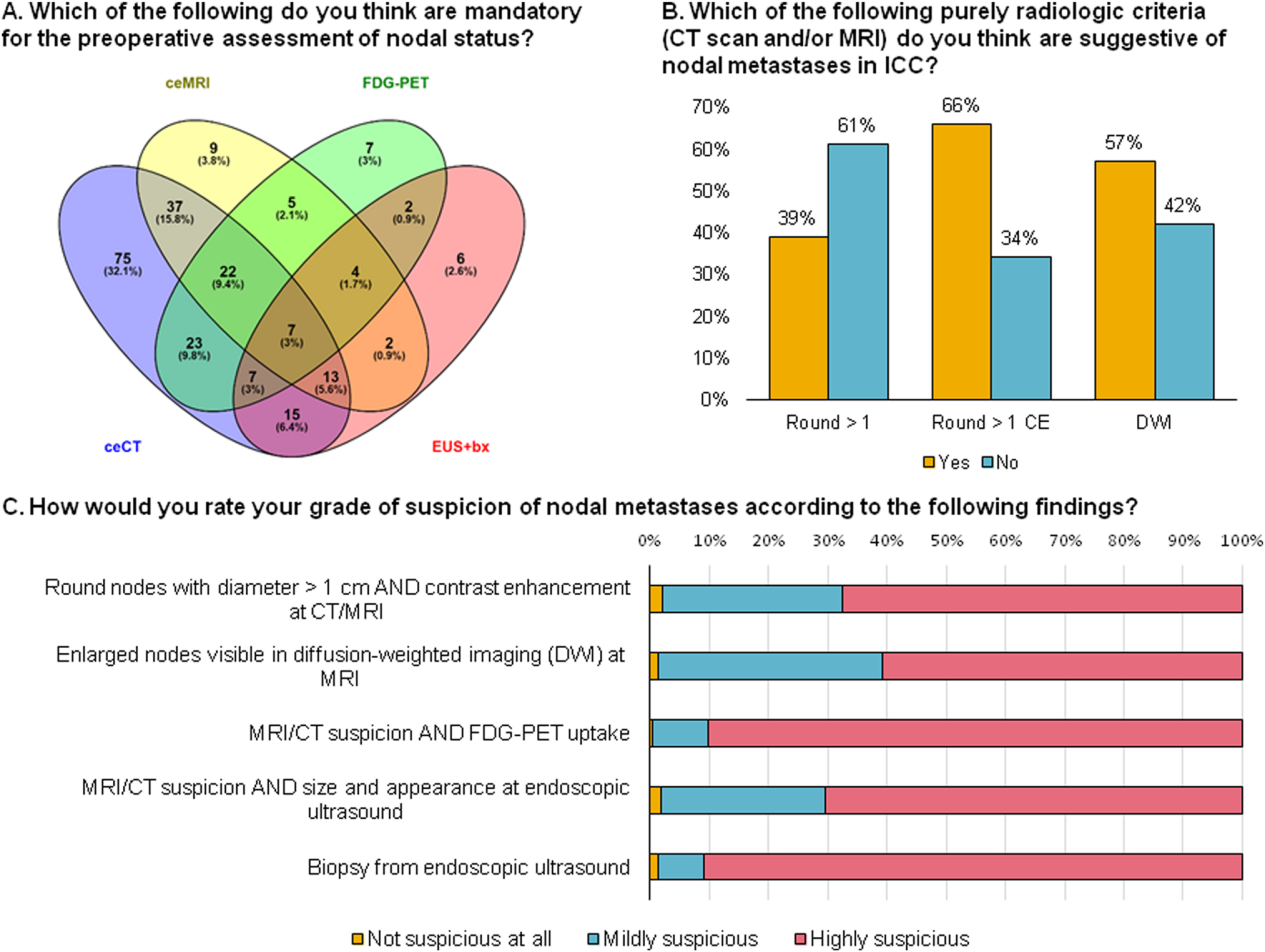
Preoperative assessment of nodal status. **A** Which of the following do you think are mandatory for the preoperative assessment of nodal status? **B** Which of the following purely radiologic criteria (CT scan and/or MRI) do you think are suggestive of nodal metastases in ICC? **C** How would you rate your grade of suspicion of nodal metastases according to the following findings? *ceMRI* contrast enhanced magnetic resonance, *ceCT* contrast enhanced computed tomography, *FDG-PET* 18F-fluoro-deoxy-glucose positron emission tomography, *EUS + bx* endoscopic ultrasound plus biopsy, *Round > 1* round nodes with diameter > 1 cm, *CE* contrast-enhanced node, *DWI* presence of nodal diffusion at diffusion weighted imaging

Preoperative evaluation of nodal status was deemed mandatory by 60 (26%), very important by 92 (39%), important by 64 (27%), slightly important by 16 (7%) and not important at all by 2 (1%) participants. Figure 1a shows the diagnostic assessment modalities which participants consider mandatory for the preoperative assessment of nodal status. More than 30% of participants consider contrast-enhanced CT scan the only mandatory imaging modality required for preoperative assessment, while 35 (15%) do not consider CT scan mandatory at all. Figure 1b shows the purely radiological criteria which the participants consider suspicious for nodal metastases. Other radiological characteristics were nodal morphology (regardless of size), necrosis, FDG-PET avidity, location along the nodal drainage of the liver, loss of hilar fat. In case of radiological suspicion 100 (43%) of participants would perform further investigations: 71 (30%) FDG-PET, 55 (23.5%) endoscopic ultrasound (EUS) with biopsy, 4 (2%) EUS alone. Figure 1c shows the grade of suspicion of nodal metastases according to different radiologic findings. Over 90% of participants deemed a positive biopsy from EUS and presence of MRI/CT suspicion with concomitant FDG-PET uptake as highly suspicious for nodal metastases.

### Definition and extent of adequate lymphadenectomy

Supplementary Fig. 2a shows the different definitions of adequate lymphadenectomy. The majority of participants (76, 33%) considered retrieval of lymph-nodes of the hepatoduodenal ligament to be adequate, while 65 (28%) answered retrieval at specific nodal stations and 64 (28%) retrieval of more than 5 lymph nodes. Only ten (4%) considered sampling of suspicious lymph nodes as adequate. Supplementary Fig. 2b shows the nodal stations that participants retrieve when performing a nodal sampling, with the most common being the stations 12a, 12b, 12p, 8a, 8p, and 5.

### Perioperative management

The final section of the survey was dedicated to questions regarding perioperative management, including indications to neoadjuvant chemotherapy and lymphadenectomy.

**Fig. 2:**
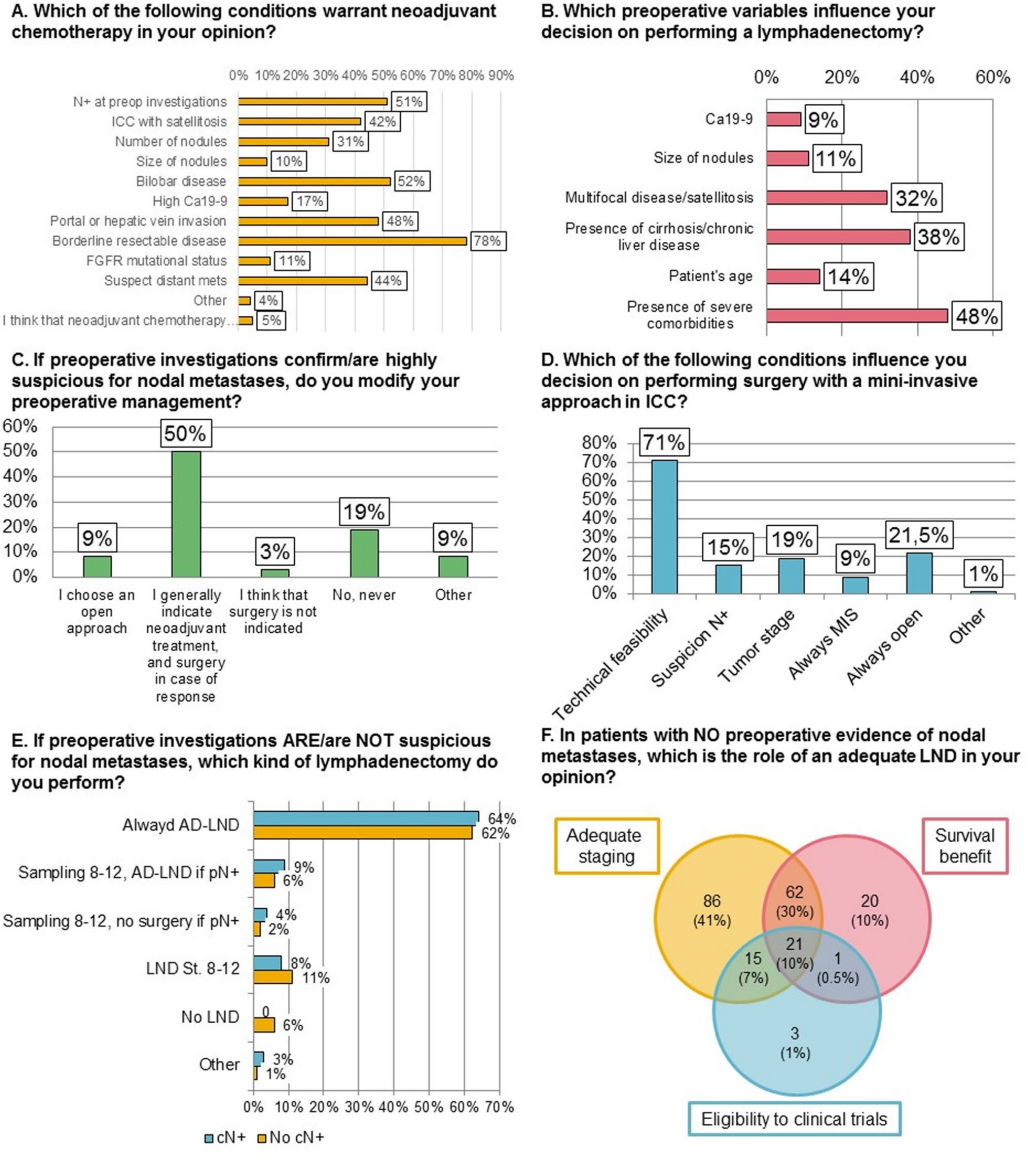
Perioperative management. **A** Which of the following conditions warrant neoadjuvant chemotherapy in your opinion? **B** Which preoperative variables influence your decision on performing a lymphadenectomy? **C** If preoperative investigations confirm/are highly suspicious for nodal metastases, do you modify your preoperative management? **D** Which of the following conditions influence you decision on performing surgery with a mini-invasive approach in ICC? **E** If preoperative investigations ARE/are NOT suspicious for nodal metastases, which kind of lymphadenectomy do you perform? **F** In patients with NO preoperative evidence of nodal metastases, which is the role of an adequate lymphadenectomy in your opinion? *LND* lymphadenectomy, *MIS* minimally invasive surgery, *AD–LND* adequate lymphadenectomy

Conditions that warrant neoadjuvant chemotherapy according to the participants (*n* = 211) are reported in Fig. 2a. The only condition on which the vast majority of participants (78%) agreed was borderline resectable disease, followed by bilobar disease (52%), nodal positivity at preoperative investigations (51%), and portal or hepatic vein invasion (48%). For the 31% who answered “number of nodules”, the median number of nodules after which they would consider neoadjuvant chemotherapy was 2 (IQR 2–3). For the 17% who answered “high CA19-9”, the median CA19-9 level after which they would consider neoadjuvant chemotherapy was 400 (IQR 200–500). For the 10% who answered “size of nodules”, the median size of nodules after which they would consider neoadjuvant chemotherapy was 5 cm (IQR 5–7).

Figure 2b illustrates preoperative variables that influence decision-making regarding lymphadenectomy. More than a third of participants (*n* = 209) considered presence of severe comorbidites (48%), presence of cirrhosis/chronic liver disease (38%) and multifocal disease/satellitosis (32%) as preoperative variables. For the 14% who answered “patient’s age”, the median age that would influence their decision-making regarding lymphadenectomy was 80 (IQR 75–80). For the 11% who answered “size of nodules”, the median size that would influence their decision-making regarding lymphadenectomy was 5 cm (IQR 3–5). For the 9% who answered “Ca19-9”, the median Ca19-9 level that would influence their decision-making regarding lymphadenectomy was 200 (IQR 100–400).

The majority of participants (81%) stated that they would somehow modify their perioperative management in case of either positive or highly suspicious preoperative investigations for nodal metastases (Fig. 2c). Half of the participants would indicate neoadjuvant treatment, with surgery in case of response, while 3% considered surgery not indicated in this case. Of the 9% who answered “other”, the majority answered that their decision-making would depend on the location of the positive/suspicious nodes.

Figure 2d illustrates which conditions would influence the decision to adopt a minimally invasive approach. Technical feasibility was the most reported factor (71%), while 21.5% of participants reported they always use an open approach.

Figure 2e shows the difference intraoperative strategies regarding lymphadenectomy according to preoperative investigations. The percentage of participants that always perform adequate lymphadenectomy was 64% in case of clinically node positive (cN +) and 62% in clinically node negative (cN0) patients, while 6% reported they perform no lymphadenectomy in case of cN0.

The participants’ opinions on the role of adequate lymphadenectomy in case of no preoperative evidence of nodal metastases are shown in Fig. 2f. The majority of participants (88%) reported adequate staging as a role of adequate lymphadenectomy: this was considered the only role in 41% cases, in conjunction with survival benefit in 30% cases, in conjunction with eligibility to clinical trials in 7% cases, and in conjunction with both survival benefit and eligibility to clinical trials in 10% cases. In Table 2**,** we report the extent of adequate lymphadenectomy stratified according to the perceived role of lymphadenectomy.

**Table 2:** Extent of lymphadenectomy performed according to the perceived role of lymphadenectomy

	Adequate staging (*n* = 184)	Survival benefit (*n* = 104)	Eligibility to clinical trials (*n* = 40)
Sampling of suspicious LN	64.2%	21.8%	14.0%
Retrieval of > 5 LN	54.3%	35.4%	10.2%
Hepatoduodenal ligament	57.7%	29.8%	12.5%
Specific nodal stations	53.6%	31.3%	15.1%

### East vs west

Twenty-seven surgeons from Eastern countries were compared to 198 surgeons from Western countries. The two groups had similar percentages of surgeons from high volume centers. Regarding preoperative staging, the Eastern group more frequently reported that FDG-PET was mandatory (48% vs 31%, *p* = 0.08) and would perform further investigations in case of radiological suspicion of N + (63% vs 40%, *p* = 0.037). The Eastern group more frequently defined adequate lymphadenectomy as sampling of suspicious nodes (19% vs 3%) and retrieval of LNs of the hepatoduodenal ligaments (42% vs 32%), than as > 5 LNs (19% vs 30%) or from more stations (19% vs 28%), *p* = 0.002. The Western group was more likely to include in AD-LND station 5 (58% vs 35%, *p* = 0.034), while the Eastern group station 7 (35% vs 16%, *p* = 0.030) and station 13 (54% vs 35%, *p* = 0.082). More participants in the Eastern group reported that LND should be performed according to tumor location (77% vs 48%, *p* = 0.007). Regarding perioperative treatment, the Western group more frequently would perform neoadjuvant therapy in case of bilobar disease (54% vs 28%, *p* = 0.018) and high CA19-9 (18% vs 4%, *p* = 0.087). More participants in the Eastern group reported that presence of cirrhosis/chronic liver disease influences their decision to perform a lymphadenectomy (40% vs 10%, *p* = 0.0004) and that their decision to perform surgery with a minimally invasive approach is influenced by the suspicion of nodal metastases (32% vs 13%, *p* = 0.032) and tumor stage (32% vs 16%, *p* = 0.093).

### High volume vs low volume

One-hundred and ten (47%) participants from high volume centers (HV group) were compared with 124 (53%) surgeons from low-volume centers (LV group). Regarding preoperative staging, the HV group more frequently reported that ceCT was mandatory (90% vs 81%, *p* = 0.06), while EUS + biopsy was more frequently mandatory for the LV group (30% vs 17%, *p* = 0.031). Regarding perioperative treatment, the HV group more frequently would perform neoadjuvant therapy in case of multifocal ICC (37% vs 26%, *p* = 0.076), according to size of the largest nodule (15% vs 5.5%, *p* = 0.037) and in case of bilobar disease (60% vs 44%, *p* = 0.027). More participants in the LV group thought that neoadjuvant chemotherapy is never warranted (9% vs 1%, *p* = 0.01). More participants in the HV group reported that presence of cirrhosis/chronic liver disease influences their decision to perform a lymphadenectomy (47% vs 30%, *p* = 0.015) and that their decision to perform surgery with a minimally invasive approach is influenced by technical feasibility (80% vs 63%, *p* = 0.009). More surgeons in the LV group reported they always adopt a minimally-invasive approach (28% vs 15%, *p* = 0.019).

### Perceived importance of nodal staging

Surgeons who gave high importance to nodal staging (HI group, *n* = 152, 65%) were compared with surgeons who gave intermediate/low important to nodal staging (ILI group, *n* = 82, 35%). Regarding preoperative staging, the HI group more frequently reported that ceMRI (49% vs 30%, *p* = 0.008) and FDG-PET (39% vs 22%, *p* = 0.009), and EUS + biopsy (28% vs 16%, *p* = 0.032) were mandatory, and would more frequently perform further investigations in case of radiological suspicion of N + (50% vs 29%, *p* = 0.002). The HI group was more likely to include station 13 (43% vs 28%, *p* = 0.029) in adequate LND. Regarding perioperative treatment, the HI group more frequently would perform neoadjuvant therapy in case of nodal positivity at preoperative investigations (62% vs 31%, *p* < 0.001), portal or hepatic vein invasion (52.5% vs 39%, *p* = 0.081). More participants in the ILI group thought that neoadjuvant chemotherapy is never warranted (10% vs 3%, *p* = 0.048). More participants in the HI group reported that patient’s age influences their decision to perform a lymphadenectomy (19% vs 11%, *p* = 0.097) and that they would recommend neoadjuvant treatment followed by surgery in case of response if preoperative investigations are highly suspicious for nodal disease (72% vs 28%, *p* = 0.003). Participants in the HI group more frequently reported that their decision to perform surgery with a minimally invasive approach is influenced by the suspicion of nodal metastases (19% vs 8%, *p* = 0.044). In case of preoperative investigations not suspicious for N +, more surgeons in the HI group reported they always perform adequate lymphadenectomy (76% vs 61%, *p* = 0.053).

## Discussion

Our survey on current approaches to lymphadenectomy in curative-intent surgical resection of intrahepatic cholangiocarcinoma shows remarkable heterogeneity in how preoperative nodal staging is carried out, how adequate lymphadenectomy is defined and in which cases it is performed.

Participants to the survey were a minority of the overall population of surgeons who received the questionnaire, however, 47% came from high volume centers for ICC, suggesting a reliable level of expertise in the sampled population.

The first part of the survey regarded preoperative staging. The preoperative assessment of nodal status was deemed important in most cases, however, 97 (41%) surgeons used a single imaging modality for preoperative nodal assessment. Most centers rely on CT scan for the assessment of nodal status, while despite its potential advantages EUS is poorly utilized. Only 43% of participants further investigates nodal status in case of radiological suspicion. This is also reflected by the fact that cN + status changes ICC management in a minority of cases, as shown in Fig. 2c. FDG-PET as a routine investigation does not seem to be widely utilized, despite evidence that it increases the diagnostic accuracy for nodal metastases [18, 19]. In accordance to the literature, though, PET positivity is regarded as the most reliable imaging modality to confirm the preoperative suspicion of nodal metastases.

One of the most interesting findings is the heterogeneous definition of what surgeons consider as “adequate” lymphadenectomy. The definition of adequacy is likely linked to the perceived aim of LND, thus we stratified responders accordingly, as shown in Table 2. Those who regard accurate staging as the aim of LND are more likely to consider sampling of suspicious LNs as adequate, differently from those for whom the aim is survival benefit, who more often consider retrieval of > 5 LNs as adequate. This is easily understood: if accurate staging is the aim, then sampling can be considered sufficient, while in case of survival benefit it is important to retrieve the minimum number of LNs as recommended by AJCC guidelines. Similar to adequacy, no consensus was reached for the nodal stations to be retrieved when performing a nodal sampling, with a maximum of 91% agreement for station 12a.

Around half of the participants stated they would recommend neoadjuvant chemotherapy in case of nodal positivity at preoperative investigations, while only 3% would not indicate surgery in such a case. This clearly reflects the perceived prognostic impact of nodal metastases on patients’ prognosis. Although extensive literature [2, 20, 21] reports a worsened prognosis in case of nodal metastases to date no evidence exists on the impact of neoadjuvant CT on this subgroup of patients. Participants who recommend neoadjuvant CT for nodal metastases tend to perform more than one imaging modality during the preoperative assessment compared to those who would not recommend CT, highlighting the correlation between the comprehensiveness of the preoperative evaluation and subsequent clinical decision-making.

Preoperative nodal status does not influence the indication to a laparoscopic approach, reflecting the tendency to perform MIS in ICC if technically feasible [22–24]. MIS for ICC has been found to be safe and feasible [24] when performed in high volume centers [24], although it seems to be associated with lower lymphadenectomy rates and harvested lymph nodes than open surgery. Indeed, 21.5% of surgeons in our survey reported they always use an open approach for ICC, but this picture might rapidly change with the increasing use of a robotic approach [25].

In addition, the presence or absence of clinically suspicious nodes has little impact on the extension of lymphadenectomy. Most participants perform an adequate LND in all cases regardless of clinical nodal status, while only a small proportion performs a nodal sampling and decides the management according to frozen sections.

The value of LND is perceived as solely related to accurate staging by 41% of participants, while 50.5% believe that LND is associated to a survival benefit. This reflects the highly debated issue of the role of LND in surgical oncology in general. While LND has long been part of the gold standard surgical treatment for several tumors, such as gastric and colon cancer [26], their extent is still occasionally under debate and a definitive survival benefit of LND per se has not been proven in several diseases. Extended lymphadenectomies beyond the gold standard have also often been found to be futile [27–29]. Thus, the absence of a consensus on the adequate extent of LND for ICC, together with the technical difficulty of LND in the hepatic hilar region and potentially increased morbidity, may discourage surgeons. In addition, although accurate staging is necessary as a prognostic tool and should still be considered reason enough to perform LND, this may have little impact on the postoperative clinical management considering that adjuvant chemotherapy should be administered at any stage after curative surgery for ICC [30]. This may further dissuade those surgeons who do not consider LND as therapeutically relevant from performing it.

In line with literature, the most common causes for not performing a lymphadenectomy were the presence of comorbidities and cirrhosis, since these conditions are related to a higher risk of developing postoperative complications [14]. However, over a third of participants report that tumor extension might influence their decision on performing LND. Indeed, a higher tumor burden increases the likelihood that other factors other than nodal metastases have an influence on long-term prognosis [31], which may render LND pointless.

Differences in LND between Eastern and Western surgeons have already been observed for gastric and rectal cancer [32, 33]. A study by Zhang et al. in 2018 [34] reported that LND for ICC was less performed in Eastern countries, although this difference disappeared in the multivariable analysis when controlling for covariates. This is partially reflected by our findings, in which, in case of preoperative suspicion of nodal metastases, surgeons from the Eastern group were more likely to perform nodal sampling followed by LND if the sampling was positive, while Western surgeons were more likely to always perform adequate LND.

Regarding center volume, surgeons from high volume centers appeared to indicate neoadjuvant therapy more often than low volume centers, with a significantly higher prevalence in several indications (multifocal ICC, large nodule size, bilobar disease). This is in line with the results of Kommalapati et al. [35] who, in an analysis of 11,344 patients treated at 1106 US facilities based on the National Cancer Database, found that patients with ICC were more likely to receive neoadjuvant therapy in high volume versus low volume centers. In our survey, surgeons from high volume centers were also less likely to report they always adopt a minimally-invasive approach, which may reflect a higher complexity of cases operated at these centers. An analysis on the type of the minimally invasive approach adopted (i.e. laparoscopic vs. robotic) was behind the scope of this study. Recent studies highlight an increased nodal yield with the mini-invasive technique, particularly by robotic approach, and this might impact on timing and possibility of adjuvant treatments [36].

Finally, the perceived importance of nodal staging had a significant impact on preoperative and perioperative management. Surgeons who gave high importance to nodal staging were more likely to perform a more thorough assessment, with more diagnostic exams and further investigations in case of suspicion of nodal metastases. They were also more likely to recommend neoadjuvant chemotherapy in case of suspicion of nodal metastases, followed by surgery in case of response.

This study has some limitations. Firstly, the survey was disseminated to 2087 surgeons, yielding a response rate of only 11.2% distributed prevalently in Western countries. The reasons for such a small response rate are probably attributable to the excessive number of surveys distributed worldwide, and the consequent disaffection of surgeons to this kind of instrument. Response rates could have been partially improved by further email re-calls or advertisement of the survey on socials, something that was intentionally not done in order not to excessively burden members of the scientific societies. However, despite its modest size, the data provides a snapshot of the current decision-making processes among this group of surgeons. Moreover since the survey is non-anonymized, it is at least free from duplicate responses. Secondly, there is a notable predominance of academic surgeons, which might be attributed to potential selection bias resulting from the involvement of (inter)national scientific societies. Lastly, it is important to note that responses reflect individual preferences and perceptions (response bias), which have not been corroborated by patient data.

In conclusion, our survey shows the great heterogeneity in the evaluation of the role and extent of lymphadenectomy for ICC, and how this is related to the perception of the importance and clinical impact of LND itself. It is evident that lymphadenectomy for ICC warrants prospective studies to provide reliable and consistent guidelines that can be shared by the HPB community for the treatment of these patients.

## Supplementary Information

Below is the link to the electronic supplementary material.

Supplementary file1 (DOCX 287 KB)

## Data Availability

The data, code and other materials are available from the corresponding author upon request.
