# Supplementary material for: A snapshot on current approaches to lymphadenectomy in liver resection for intrahepatic cholangiocarcinoma: results from an international survey
Source: Updates Surg. 2024 May 7;76(5):1797–805. doi: 10.1007/s13304-024-01852-0 (PMC11455682; doi:10.1007/s13304-024-01852-0)
Supplement: Supplementary file 1 — Supplementary file1 (DOCX 287 KB) [file 13304_2024_1852_MOESM1_ESM.docx]

**Supplementary Figure 1.** Country of origin of the participants


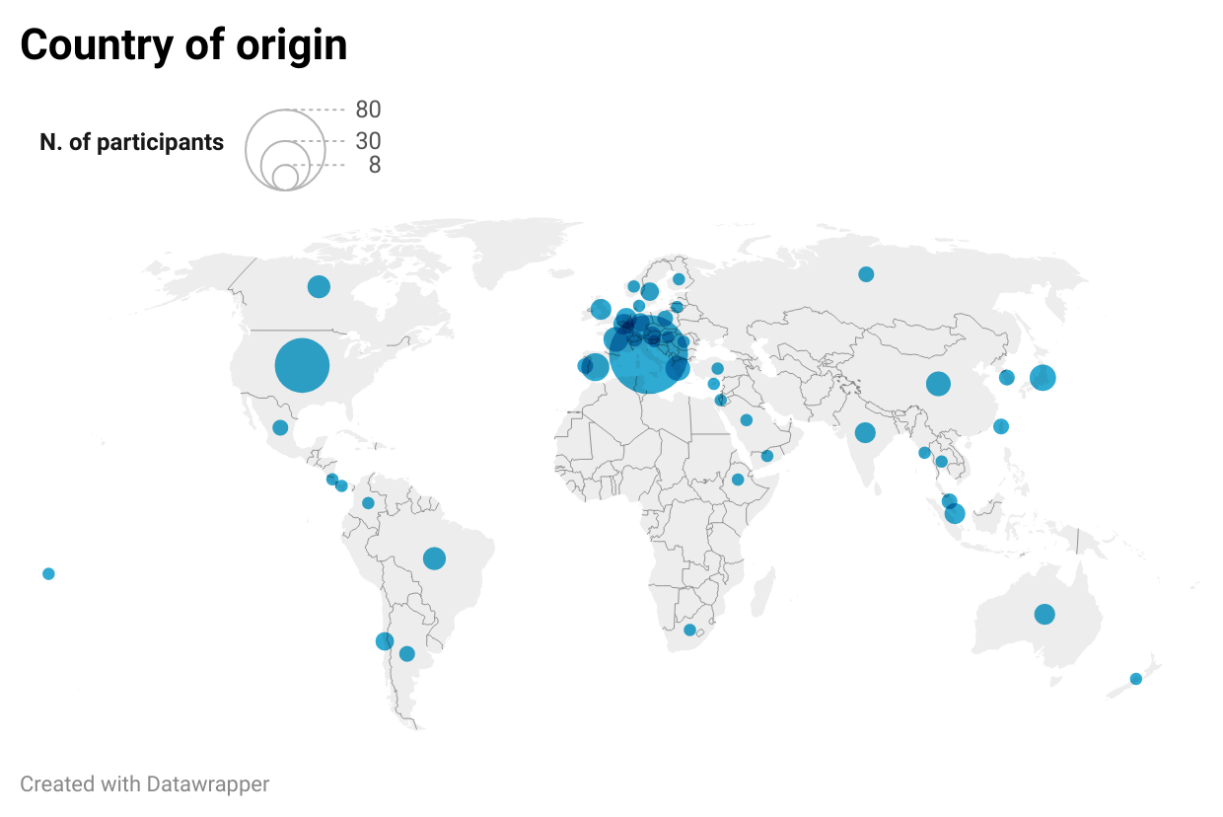


**Supplementary Figure 2.** Definition of adequate lymphadenectomy.


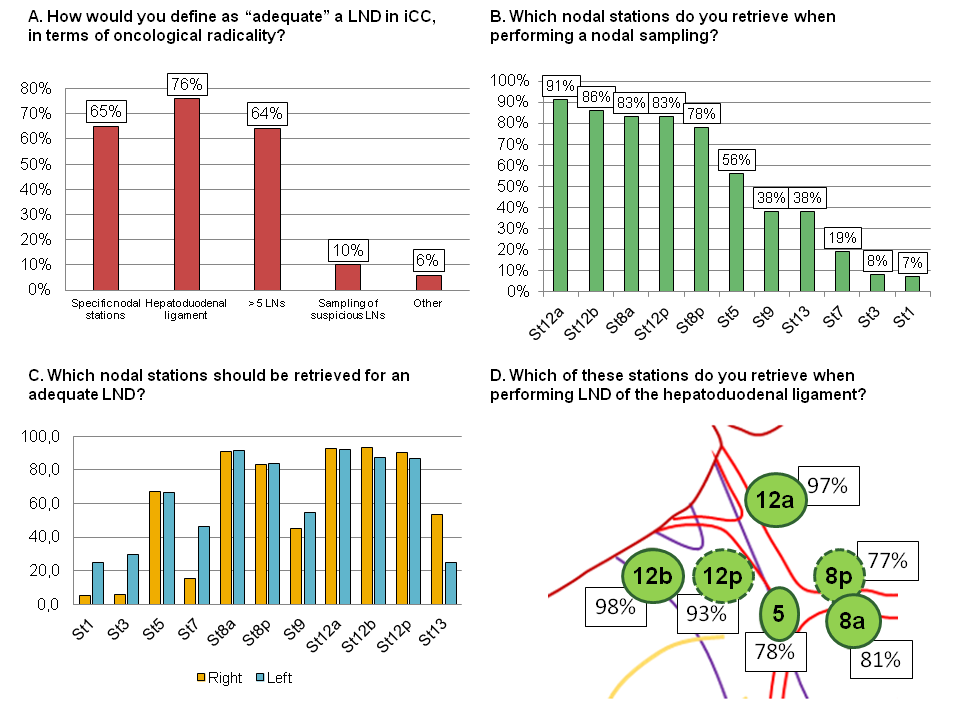

A) How would you define as “adequate” a lymphadenectomy in ICC, in terms of oncological radicality? B) Which nodal stations do you retrieve when performing a nodal sampling? C) Which nodal stations should be retrieved for an adequate lymphadenectomy? D) Which of these nodal stations do you retrieve when performing lymphadenectomy of the hepatoduodenal ligament?

ICC = intrahepatic cholangiocarcinoma; LND = lymphadenectomy
